# A glimpse into the biogeography, seasonality, and ecological functions of arctic marine *Oomycota*

**DOI:** 10.1186/s43008-019-0006-6

**Published:** 2019-06-20

**Authors:** Brandon T. Hassett, Marco Thines, Anthony Buaya, Sebastian Ploch, R. Gradinger

**Affiliations:** 1 UiT-Norges arktiske universitet, BFE, NFH bygget, Framstredet 6, 9019 Tromsø, Norway; 2 Senckenberg Biodiversity and Climate Research Centre, Senckenberganlage 25, 60325 Frankfurt am Main, Germany; 3 Department of Biological Sciences, Goethe University, Institute of Ecology, Evolution and Diversity, Max-von-Laue-Str. 13, 60435 Frankfurt am Main, Germany; 4 Translational Biodiversity Genomics Centre, Georg-Voigt-Str. 14-16, 60325 Frankfurt am Main, Germany

**Keywords:** Biodiversity, 18S, Diatom parasites, GeoChip, Sea ice, Sediment

## Abstract

High-latitude environments are warming, leading to changes in biological diversity patterns of taxa. *Oomycota* are a group of fungal-like organisms that comprise a major clade of eukaryotic life and are parasites of fish, agricultural crops, and algae. The diversity, functionality, and distribution of these organisms are essentially unknown in the Arctic marine environment. Thus, it was our aim to conduct a first screening, using a functional gene assay and high-throughput sequencing of two gene regions within the 18S rRNA locus to examine the diversity, richness, and phylogeny of marine *Oomycota* within Arctic sediment, seawater, and sea ice. We detected *Oomycota* at every site sampled and identified regionally localized taxa, as well as taxa that existed in both Alaska and Svalbard. While the recently described diatom parasite *Miracula helgolandica* made up about 50% of the oomycete reads found, many lineages were observed that could not be assigned to known species, including several that clustered with another recently described diatom parasite, *Olpidiopsis drebesii*. Across the Arctic, *Oomycota* comprised a maximum of 6% of the entire eukaryotic microbial community in Barrow, Alaska May sediment and 10% in sea ice near the Svalbard archipelago. We found Arctic marine *Oomycota* encode numerous genes involved in parasitism and carbon cycling processes. Ultimately, these data suggest that Arctic marine *Oomycota* are a reservoir of uncharacterized biodiversity, the majority of which are probably parasites of diatoms, while others might cryptically cycle carbon or interface other unknown ecological processes. As the Arctic continues to warm, lower-latitude *Oomycota* might migrate into the Arctic Ocean and parasitize non-coevolved hosts, leading to incalculable shifts in the primary producer community.

**Electronic supplementary material:**

The online version of this article (10.1186/s43008-019-0006-6) contains supplementary material, which is available to authorized users.

## INTRODUCTION

The Arctic is warming at a rapid rate. Elevated atmospheric temperatures and the inflow of warmer waters from the Pacific and Atlantic oceans are reducing sea ice extent and thickness (Vihma 2014). The associated physical changes in the Arctic marine environment are altering the phenology of primary producers (Castellani et al. 2017), their associated consumers, and subsequent higher trophic levels (Feng et al. 2018). As sea surface temperatures continue to increase, southerly Atlantic and Pacific species are migrating north, ushering in novel biological interactions that have unknown consequences on existing Arctic marine food webs (Kortsch et al. 2015). The Arctic Ocean remains one of the least-studied oceanographic regions in the world, with large gaps remaining in the current biodiversity inventory, specifically for microbes. While microbes are estimated to comprise > 90% of all oceanic biomass (Suttle 2007), their activity has yet to be fully integrated into Arctic marine ecology.

Heterotrophic eukaryotic microbes (HEMs), primarily fungi and fungal-like organisms, are known saprotrophs and parasites in freshwater and marine ecosystems (Sparrow 1960; Johnson and Sparrow 1970). Convergent morphology, taxonomy in-flux, and difficulties in cultivation associated with Arctic marine HEMs hinder the identification, characterization, and subsequent integration of their activity into marine systems ecology, especially holistic modeling efforts. As a result, the diversity and distribution of select Arctic marine HEMs is uncharacterized, unreported, or knowingly excluded in published assessments of unicellular eukaryotic biodiversity (Poulin et al. 2011), resulting in a general reductionist understanding of their ecological contributions (Keeling and del Campo 2017).

*Oomycota* are globally distributed zoosporic heterokonts, previously considered members of the kingdom *Fungi,* that are now known to phylogenetically branch within the *Straminipila*-*Alveolata*-*Rhizaria* superkingdom (Burki and Keeling 2014). *Oomycota* have cell walls comprised of cellulose derivatives that serve as structural components, as opposed to chitin in true *Fungi* (Thines 2018). *Oomycota* are a genetically and morphologically diverse clade that contains at least 1500 species in 100 genera that can form hyphae or exist as simple holocarpic thalli (Beakes and Thines 2017). Members of *Oomycota* are known pathogens of nematodes (Phillips et al. 2008), zooplankton (Thomas et al. 2010), micro-algae (Thines et al. 2015, Buaya et al. 2017), and fish (van West 2006). Some diatom-associated *Oomycota*_s_ have been reported from subarctic marine environments (Hanic et al. 2009; Scholz et al. 2014, 2016), but sparingly from the Arctic Ocean. Specifically, *Oomycota* have been observed as parasites on algae in the Canadian Arctic (Küpper et al. 2016) and have been reported on marine bird feathers in Svalbard (Singh et al. 2016). Establishing a current inventory of *Oomycota* appears important and urgent due to the lack of a current baseline and the ongoing northward movement of Atlantic and Pacific species. This migration will lead to novel encounters between parasites and non-coevolved hosts.

To establish a baseline of *Oomycota* diversity, abundance, and distribution across the Arctic, we conducted high-throughput sequencing of the hypervariable V3–4 and V9 regions of the 18S rRNA gene from sea ice, seawater, and sediment samples across the western Arctic. We supplemented these analyses with a functional gene survey from under-ice Alaskan sediment. We hypothesized that marine *Oomycota* are widely distributed across the Arctic and encode uncharacterized genetic diversity that facilitates biogeochemical cycling and the turnover of biological material.

## MATERIALS AND METHODS

### Environmental sampling

**Fig. 1:**
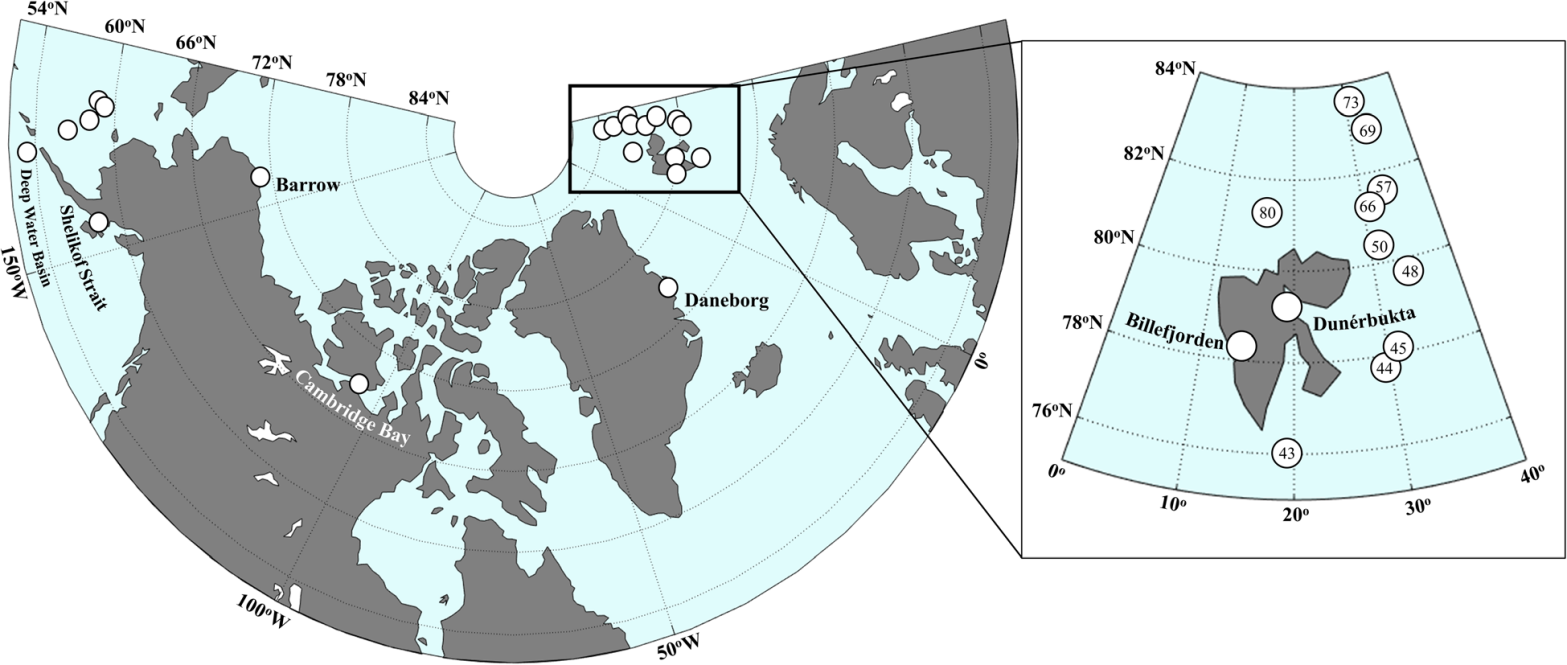
Sampling sites of sea ice, seawater, and sediment across the Arctic, including the Bering Sea, Greenland and Svalbard. Note that Barrow, Alaska has been sampled several times (see Table 1)

Sea ice, water column and under-ice sediment samples were collected across the Arctic and Bering Sea between 2014 and 2017 (Table 1, Fig. 1) onboard the R/V Polarstern, R/V Sikuliaq, and from snowmobile in the coastal sea ice environments in Alaska, Greenland, and Svalbard. Seawater was collected using a CTD/Rosette sampler in 10 L Niskin bottles. At least 1 liter of seawater was collected to sample the suspended community, which was subsequently vacuum-filtered onto 47 mm, 0.2 μm nuclepore filters (Sartorius, Göttingen, Germany) for high-throughput sequencing. Additional samples were screened with a light microscope (Carl Zeiss, Oberkochen, Germany), and photographed using a digital camera (Carl Zeiss, Oberkochen, Germany). Ice cores were extracted at each sea ice station using a 9 cm Kovacs ice corer. The bottom 10 cm of each core was sectioned using an ethanol-sterilized handsaw. Ice core sections were melted at room temperature with the addition of 1 L of 0.22 μm-filtered seawater. After complete melting of the ice cores, samples were vacuum-filtered onto 0.2 μm filters. After filtration, all filters were immediately stored in sterile polypropylene tubes at − 80 °C and kept frozen in the dark until analysis. Sediment traps with 72 mm diameter and 1.8 L volume (Model 28.xxx series, KC-Denmark, Silkeborg, Denmark) were deployed at 5 and 20 m for 8 h and 37 min at a single ice station (station 80). Sediment samples were collected in Barrow, Alaska in triplicate in May and June of 2014 using a ponar grab that was deployed through a hole in the ice. Sediment was stored in sterile polypropylene tubes at − 80 °C until DNA extraction.

**Table 1:** Metadata sheet of sites sampled and analyzed for *Oomycota*

Station	Date	Location	Depth (m)	^o^C	Salinity	Snow depth (cm)	Notes
Barrow, Alaska	13-Jan-14	N71.365 W156.538	–	–	–	23.5	Sea ice
Barrow, Alaska	13-Jan-14	N71.365 W156.538	10	− 1.8	31.7	–	Sediment
Barrow, Alaska	10-Mar-14	N71.365 W156.538	–	–	–	12.5	Sea ice
Barrow, Alaska	10-Mar-14	N71.365 W156.538	10	−1.8	31.7	–	Sediment
Barrow, Alaska	9-Apr-14	N71.365 W156.538	–	–	–	12.8	Sea ice
Barrow, Alaska	9-Apr-14	N71.365 W156.538	10	−1.8	31.7	–	Sediment
Billefjorden, Svalbard	26-Apr-14	N78.660 E16.730	–	–	–	3.0	Sea ice
Dunérbukta, Svalbard	4-May-14	N78.190 E18.830	–	–	–	14.0	Sea ice
Barrow, Alaska	28-May-14	N71.365 W156.538	–	− 1.8	–	–	Sea ice
Barrow, Alaska	28-May-14	N71.365 W156.538	10	−1.8	31.7	–	Sediment
Barrow, Alaska	15-Jun-14	N71.365 W156.538	–	−1.8	–	6.0	Sea ice
Barrow, Alaska	15-Jun-14	N71.365 W156.538	10	−1.8	31.7	–	Sediment
Daneborg 6–19, Greenland	19-Jun-14	N74.300 W20.340	–	–	–	26.0	Sea ice
Cambridge Bay, Canada	20-Jun-14	N69.023 W105.340	NA	–	–	0	Sea ice
Barrow, Alaska	13-Aug-14	N71.365 W156.538	10	−1.8	31.7	–	Seawater
Barrow, Alaska	13-Aug-14	N71.365 W156.538	10	−1.8	31.7	–	Sediment
Shelikof Strait, Alaska	14-Mar-15	N58.299, W153.878	225	6.2	32.5	–	Shelf
Deep water Basin, Aleutians	16-Mar-15	N53.611 W164.592	266	5.6	33.5	–	Shelf
Pribilof Islands	20-Mar-15	N56.533, W167.990	104	5.3	32.9	–	Shelf
Bering Sea Shelf	21-Mar-15	N57.878, W168.856	64	0.3	32.2	–	Shelf
Marginal Ice Zone, Bering Sea	24-Mar-15	N58.618, W170.720	72	−1.7	31.7	–	Shelf
Sea Ice Station, Bering Sea	25-Mar-15	N58.574, W170.863	–	–	–	1.0	Shelf
43	24-Jun-17	N76.178 E19.910	0.6	3.5	34.5	–	Shelf
43	24-Jun-17	N76.178 E19.910	22	2.9	34.5	–	Shelf
43	24-Jun-17	N76.178 E19.910	178	2.1	35.9	–	Shelf
44	25-Jun-17	N77.895 E30.042	0.7	−1.6	34.2	–	Shelf
44	25-Jun-17	N77.895 E30.042	35	−1.7	34.4	–	Shelf
44	25-Jun-17	N77.895 E30.042	246	−1.5	34.8	–	Shelf
45	25-Jun-17	N78.102 E30.471	–	–	–	6.2	Sea ice
48	27-Jun-17	N79.816 E34.032	1	−1.4	33.8	–	Polynya
48	27-Jun-17	N79.816 E34.032	20	−1.7	34.3	–	Polynya
48	27-Jun-17	N79.816 E34.032	269	−0.7	34.8	–	Polynya
50	28-Jun-17	N80.556 E31.207	–	–	–	7.6	Sea ice
57	30-Jun-17	N81.745 E32.941	1	−1.7	33.9	–	Shelf slope
57	30-Jun-17	N81.745 E32.941	36	−1.6	34	–	Shelf slope
57	30-Jun-17	N81.745 E32.941	1979	−0.6	34.9	–	Shelf slope
66	2-Jul-17	N81.650 E32.455	–	–	–	3.6	Sea ice
69	5-Jul-17	N83.029 E33.208	2	−1.7	34		Basin
69	5-Jul-17	N83.029 E33.208	25	−1.7	34.1		Basin
69	5-Jul-17	N83.029E33.208	3652	−0.6	34.9		Basin
73	7-Jul-17	N83.6645 E31.7700	–	–	–	3.5	Sea ice
80	12-Jul-17	N81.326 E16.934	1	−1.3	32.8	–	Shelf slope
80	12-Jul-17	N81.326 E16.934	21	−0.9	33	–	Shelf slope
80	12-Jul-17	N81.326 E16.934	967	3.6	35	–	Shelf slope
80	12-Jul-17	N81.326 E16.934	5	−1.4	32.8	–	Sed. trap
80	12-Jul-17	N81.326 E16.934	20	−1.3	32.8	–	Sed. trap

### DNA extraction and sequence processing

DNA was extracted and PCR-amplified, as previously described (Hassett and Gradinger 2016; Hassett et al. 2017). Briefly, we used primers that target three separate hypervariable regions of the 18S rRNA gene. We generated ~ 400 base pair sequences from the V3-V4 regions using the 18S-82F (5′-GAAACTGCGAATGGCTC-3′) and Ek-516R (5′-ACCAGACTTGCCCTCC-3′) primer pair. This primer pair was used primarily to deep-sequence (six samples per MiSeq run) samples from Barrow, Alaska, plus one sample from Svalbard and to obtain sequences informative enough for phylogenetic inference. To supplement this analysis, we generated ~ 170 base pair sequences from the V9 region using the Euk1391f: (5′- GTACACACCGCCCGTC-3′) and EukBr: (5′- TGATCCTTCTGCAGGTTCACCTAC-3′). This primer pair was used for spatial analysis. PCR products were generated using fusion primers with the Fluidigm CS1 or CS2 universal oligomers added to 5′ ends. Secondary PCR and sequencing was performed at Michigan State University’s Genomics Core Lab. Secondary PCR was conducted with dual-indexed, Illumina-compatible primers to complete library construction. Final PCR products were batch-normalized using an Invitrogen SequalPrep DNA Normalization plate and products recovered from the plate were then pooled. The pool was quantified using a combination of Qubit dsDNA HS, Agilent Bioanalyzer DNA 1000, and Kapa Illumina Library Quantification qPCR assays. The pool was loaded onto two (i.e. sequenced twice to increase sequencing depth) standard MiSeq v2 flow cells and sequencing was performed in a 2x250bp paired-end format using MiSeq v2 500 cycle reagent cartridges. CPCustom primers for sequence reads one and two, as well as index read one that was complementary to the Fluidigm CS1 and CS2 oligos, were added to appropriate wells of the reagent cartridge. Base calling was done by Illumina Real Time Analysis (RTA) v1.18.54 and the output was demultiplexed and converted to FastQ format with Bcl2fastq v2.19.1. Sequence analysis and clustering was conducted using Mothur v1.33.3 (Schloss et al. 2009; Kozich et al. 2013). Sequences with ambiguous base calls were eliminated (maxambig = 0) from all datasets. Sequences were aligned using the SILVA (Quast et al. 2013) reference database (Release 123), screened for chimeras (Edgar et al. 2011) and classified with SILVA (Release 123), using the K-nearest neighbor algorithm (bootstrap cutoff value of 75% with 1,000 iterations). Sequences classified as Bacteria, Archaea, and Metazoans were removed from datasets before analysis. The remaining sequences were clustered into operational taxonomic units (OTUs) at a 97% similarity cutoff and used for further analyses.

### Functional gene survey

For functional gene analysis, DNA was extracted from triplicate under-ice sediment samples from Barrow, Alaska collected in May and June 2014. After extraction, DNA was pooled and analyzed using the GeoChip (He et al. 2007) functional gene microarray (GeoChip 5.0; Glomics Inc., Norman, OK). Amplification, labeling, hybridization, imaging, and data processing were conducted by the Institute for Environmental Genomics at the University of Oklahoma according to published protocols (Van Nostrand et al. 2016). Signal intensity was normalized to display all positive probes detected in each sample. Probe data were removed from the output if the signal to noise ratio was below 2 or if the signal was < 200 or < 1.3 times the background.

### Phylogeny

After OTU clustering of our V3-V4 sequences, the top 100 most-abundant *Oomycota* taxa from across the Arctic were aligned using MUSCLE as implemented in MEGA7 v7.0.26 (Kumar et al. 2016). Sequences from *Miracula helgolandica*, *Olpidiopsis drebesii*, and reference sequences obtained from NCBI by manual selection for a balanced representation of the known oomycete orders, as well as using the TrEase webserver (http:// thines-lab.senckenberg.de/trease) were added to the OTU sequences. This database was then aligned with a gap opening penalty of − 400 and a gap extension penalty of − 4. Leading and trailing sequences were end-trimmed to assure that tests of molecular phylogeny analyzed overlapping regions. The Minimum Evolution algorithm was used to test phylogenetic inference with 500 bootstraps and all values set to default, except for the selection of the Tamura-Nei substitution model. The resulting alignment is supplied as Additional file 1. Trees were visualized and edited in MEGA and exported as vector graphic for further editing.

## RESULTS

**Fig. 2:**
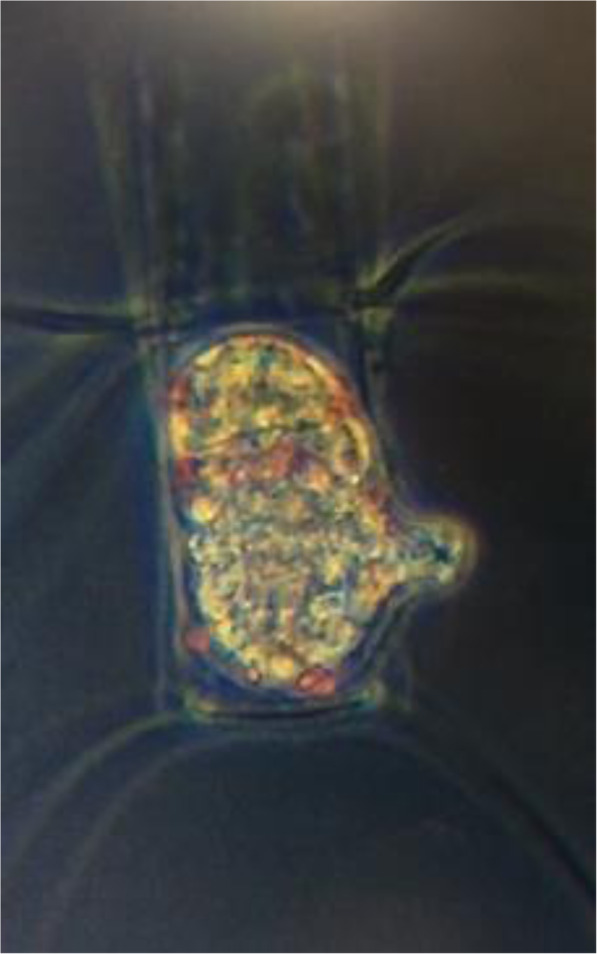
Micrograph of *Chaetocerous* sp. parasitized by an endobiotic holocarpic member of *Oomycota*

**Fig. 3:**
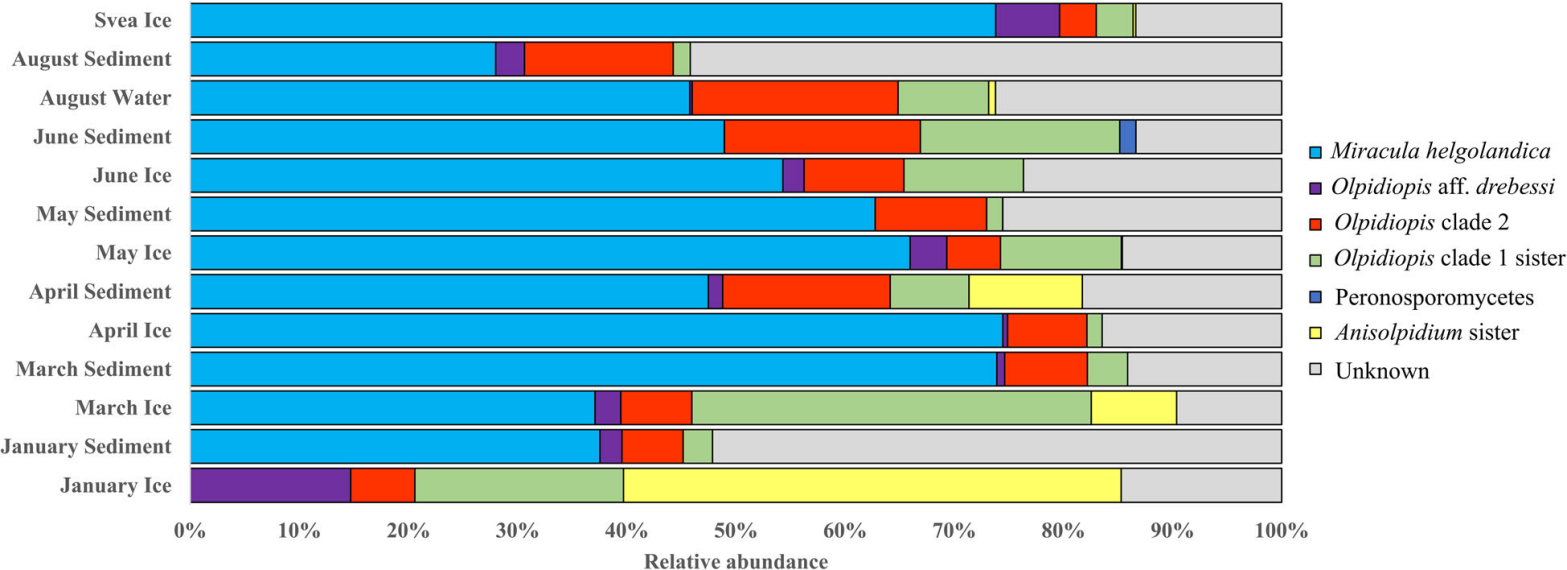
Seasonal relative abundance of the top 100 *Oomycota* V3-V4 OTUs detected in Barrow, Alaska, as well as one site in Svea, Svalbard (May of the same year). The classification scheme corresponds to phylogenetic position of 18S rRNA V3-V4 OTUs, as these sequences were unidentifiable with a Bayesian classification method

A quick screening of environmental samples revealed diatoms that were parasitized by members of *Oomycota* (Fig. 2). These observations are in-line with a relatively high proportion of oomycete reads found in the DNA sequencing approach. Specifically, after V3-V4 sequence processing and removal of prokaryote and metazoan sequences, 16,351,684 unique DNA sequence reads were retained for analysis. Of these, 290,077 (1.7%) were classified as *Oomycota*. *Oomycota* were detected in every sample, both sediment and sea ice communities, in coastal Alaskan marine environments. In these systems, the *Oomycota* generally comprised a greater proportion of total reads in sediment communities (average relative abundance (ARA) = 2.0%; standard deviation (SD) = 1.9), relative to sea ice (ARA = 0.3%; SD = 0.4). *Oomycota* contributed a maximum of 5.7% of the total eukaryotic microbial community in May sediment and a maximum of 0.9% of total eukaryotic microbial community in April sea ice. Nearly the entire community of *Oomycota* sequences (92.7%) was represented by unclassifiable sequences, while the remaining sequences were classified as *Aphanomyces*, *Aplanopsis*, *Halipththoros*, *Halocrusticida*, *Halophytophthora*, *Lagenidium*, *Leptolegnia*, *Olpidiopsis*, *Pythium*, and *Saprolegnia*. Phylogenetic inference of *Oomycota-allied DNA data* revealed that approximately 50% of the sequences corresponded to the recently-described diatom parasite *M. helgolandica*, which is not yet integrated into HTS classification databases (Fig. 3).

After V9 sequence processing and removal of prokaryotes and metazoan sequences, 16,456,575 sequences were retained for analysis. Of these, 130,186 (0.8%) sequences were classified as *Oomycota*. *Oomycota* were detected in all sites, except three chlorophyll maxima samples (sites 43, 80, Deep Water Basin). *Oomycota* had higher relative abundances in sea ice communities (ARA = 1.1%; SD = 2.5), compared to surface seawater (ARA = 0.02%; SD = 0.03), chlorophyll maxima (ARA = 0.1%; SD = 0.3), and bottom communities (ARA = 0.9%; SD = 1.9). They contributed a maximum proportion of 9.5% of the total eukaryotic microbial community from sea ice station 66. Nearly the entire community of *Oomycota* sequences (99%) were represented by unclassifiable sequences, while the remaining sequences were classified as *Halocrusticida* and *Pythium*. However, it needs to be noted that the short sequences are more difficult to phylogenetically assign and that there is, as yet, no reference sequence for available for the 18S rRNA V9 region of *M. helgodandica*. One *Oomycota* sequence was observed in the 20 m sediment traps and 28 sequences were obtained from the 5 m trap.

**Fig. 4:**
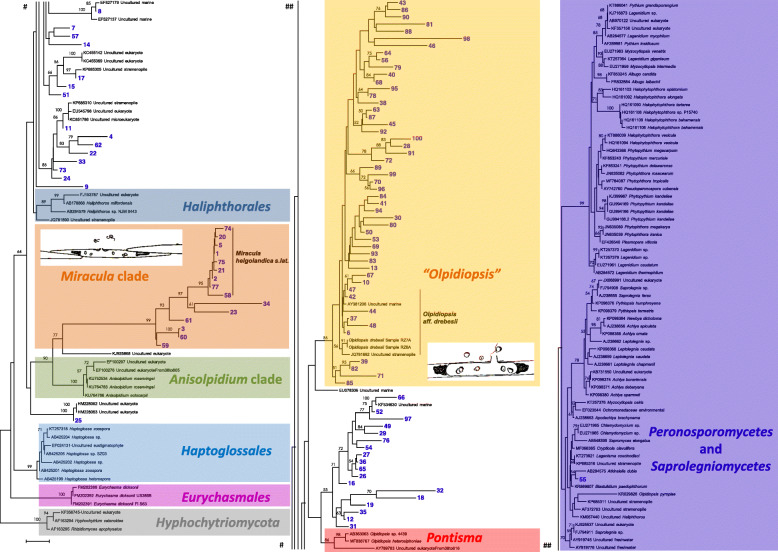
Phylogenetic tree (Minimum Evolution) based on the V3–4 regions of the nrSSU of oomycetes. Bootstrap support values > 50% are given on the branches leading to the respective nodes. The bar indicates the number of substutions per site

Operational taxonomic unit clustering of the V3-V4 region identified 36,691 distinct *Oomycota* taxa (32,294 singletons). The two most abundant V3-V4 OTUs were observed 127,754 times (42% of all *Oomycota* observations). The top-100 most abundant V3-V4 OTUs were phylogenetically analyzed. After end-trimming the V3-V4 alignment had 408 sites. The phylogenetic reconstruction revealed that the majority of our top-100 abundant OTUs were present in three major groups that branch basal to the crown oomycetes (Fig. 4). These clades represented a strongly supported group around *M. helgolandica* (a parasite of *Pseudonitzschia* diatoms) that contained the three most abundant OTUs, an unsupported, paraphyletic group around *O. heterosiphoniae* (a parasite of red algae), and a weakly supported clade around *O. drebesii* (a parasite of *Rhizosolenia* diatoms). Specifically, the top-two most abundant OTUs represent *M. helgolandica*, identified by manual curation. The third most-abundant V3-V4 OTU represents an unknown lineage of *Miracula* and was detected every month in both sea ice and under-ice sediment. Only one of the 100 most abundant OTUs clustered with the crown oomycetes and was identified as a member of the genus *Atkinsiella*, which contains holocarpic parasites of crustaceans.

Biogeographical assessment of our 18S sequences revealed a broad-distribution of many taxa across the Arctic Ocean. Specifically, comparative analysis of all *Oomycota*-classified V3-V4 OTUs from our samples revealed 52 OTUs that were found in both Svalbard and Barrow, Alaska. V9 analysis revealed that the most abundant OTU was detected in both the Gulf of Alaska, as well as Svalbard. Three V9 OTUs, comprising 17 observations, were found exclusively in the Bering Sea.

The GeoChip 5.0 contains 167,403 unique probes (many copies of the same gene derived from different species) to which environmental DNA can hybridize. Environmental DNA from Barrow, Alaska sediment hybridized to 63,403 (37%) of the available GeoChip probes. May sediment DNA hybridized to available 56,729 probes and 55,723 probes hybridized to June sediment DNA. Detected genes are characterized as being involved in biogeochemical cycling of carbon, nitrogen, sulphur, phosphorous, as well as in natural product synthesis. Genes from each taxonomic domain of Life were detected, hybridizing to: 22% of possible viral probes, 30% of eukaryotic probes, 41% of bacterial probes, and 29% of *Archaea* probes. *Oomycota* were represented by 110 probes (39% of all available *Oomycota* probes) that were derived from *Achlya*, *Aphanomyces*, *Hyaloperonospora*, *Phytophthora*, *Pythium*, and *Saprolegnia*. These hybridized *Oomycota* probes are characterized as being involved in carbon cycling, nitrate assimilation, sulphur assimilation, metal homeostasis, and virulence (Table 2). The most abundant *Oomycota* genes were pectate lyase and INF1 elicitin. Of all available probes, *Oomycota* pectate lyase was the 408th most-abundant gene detected in May sediment; and the *Oomycota*-derived INF1 elicitin gene was the 267th most-abundant gene detected in June sediment.

**Table 2:** GeoChip 5.0 probe data displaying the top-25 most abundant (proxied by probe intensity) genes in May and June sediment from Barrow, Alaska

Gene category	Probe Origin	May Signal	June Signal	Geochip gene name
Virulence	*Phytophthora ramorum*	5384.2278	3067.5303	INF1_elicitin_Oomycetes
Virulence	*Phytophthora boehmeriae*	2995.661	150.4736	INF1_elicitin_Oomycetes
Carbon cycling	*Phytophthora infestans*	2284.1703	4777.8158	pectate_lyase_Oomycetes
Virulence	*Phytophthora infestans*	2100.9598	4227.5515	PcF_Oomycetes
Carbon cycling	*Phytophthora infestans*	1271.4373	744.2255	pectate_lyase_Oomycetes
Virulence	*Phytophthora sojae*	1169.2235	1060.4361	INF1_elicitin_Oomycetes
Virulence	*Hyaloperonospora parasitica*	968.5541	764.7643	ATR13_Oomycetes
Virulence	*Phytophthora citrophthora*	919.4098	1041.0091	INF1_elicitin_Oomycetes
Virulence	*Phytophthora brassicae*	900.5573	668.7791	INF1_elicitin_Oomycetes
Virulence	*Phytophthora sojae*	822.8056	575.416	necrosis_Oomycetes
Virulence	*Phytophthora infestans*	787.8103	842.6593	AVR1_Oomycetes
Carbon cycling	*Phytophthora capsici*	758.0665	769.5996	Pg_Oomycetes
Virulence	*Phytophthora infestans*	554.6938	469.7703	serine_protease_inhibitor_Oomycetes
Virulence	*Phytophthora cinnamomi*	511.2635	861.0117	glucanase_inhibitor_Oomycetes
Carbon cycling	*Phytophthora infestans*	485.5348	344.3584	pectin_lyase_Oomycetes
Carbon cycling	*Phytophthora parasitica*	464.4053	293.4849	Pg_Oomycetes
Virulence	*Phytophthora sojae*	454.0621	375.3379	INF1_elicitin_Oomycetes
Carbon cycling	*Phytophthora sojae*	434.6085	1559.3231	mannanase
Virulence	*Phytophthora ramorum*	420.739	456.169	INF1_elicitin_Oomycetes
Carbon cycling	*Phytophthora cinnamomi*	409.9356	375.8736	Pg_Oomycetes
Carbon cycling	*Phytophthora infestans*	403.822	532.2254	pectate_lyase_Oomycetes
Virulence	*Phytophthora infestans*	397.5013	356.1048	necrosis_Oomycetes
Carbon cycling	*Phytophthora infestans*	372.6894	289.1474	chitin_synthase_protist
Carbon cycling	*Phytophthora infestans*	365.5732	589.2046	xylose_isomerase_Oomycetes
Virulence	*Phytophthora brassicae*	362.6233	242.5819	INF1_elicitin_Oomycetes

## TAXONOMY

Not applicable.

## DISCUSSION

The *Oomycota* are common members of freshwater aquatic ecosystems and terrestrial environments that interface degradation processes and establish symbiotic relationships with a variety of organisms (Thines 2014). In the marine environment, the diversity and functioning of *Oomycota* is poorly understood. However, many *Oomycota* are described as pathogens of algae (Beakes and Thines 2017, Tsirigoti et al. 2013, Li et al. 2010). In the Arctic Ocean and other high-latitude environments, reports of *Oomycota* are sparse and sporadic; consequently, the diversity, functioning, and general ecology of this important group of organisms is largely unknown.

OTU clustering and analysis revealed a broad distribution of several *Oomycota* across the Arctic Ocean, underscoring that Arctic oomycetes are both widespread and present in diverse environments. Both of our primer sets identified *Oomycota* taxa that were shared between sites in Alaska and those in Svalbard (> 5000 km distance). In general, our 18S rRNA sequencing data indicated a consistently low (< 1%) contribution of *Oomycota*-classified sequences relative to other eukaryotic microbial organisms. However, under specific environmental conditions, these proportions approached 10% of the eukaryotic microbial community. Phylogenetic analysis of the 100 most abundant V3-V4 OTUs revealed that manysequences could not be assigned to any known oomycete species. These data indicate that Arctic marine *Oomycota* are a reservoir of undescribed biodiversity, even though only the top-100 most abundant OTUs were analyzed in this study. Though many detected DNA sequences represent potentially novel lineages, most OTUs phylogenetically branched into three major groups, all of which contain species described as holocarpic pathogens of photosynthetic organisms. The most abundant oomycete OTUs that we found were closely related or conspecific with *Miracula helgolandica*, a recently-described parasitoid of *Pseudonitzschia* diatoms, known from temperate coastal waters of Canada (Hanic et al. 2009) and Germany (Buaya et al. 2017). Sequences allied to *M. helgolandica* contributed ~ 50% of all the oomycete V3-V4 dataset reads. In addition to *M. helgolandica s. lat*., at least two additional, still undescribed, species are present, one of which is represented by the 3rd most abundant V3-V4 OTU. Another major clade contained the recently described *O. drebesii* (Buaya et al. 2017), with more than a dozen independent lineages that might represent additional undescribed diatom parasitoids. The third major phylogentic group that we detected is less well-defined, but includes a parasite of red algae, rendering it tempting to speculate that the lineages found within it could also be pathogens of multicellular algae. In-line with recent studies that add evidence to the widespread presence of oomycete parasitoids in marine plankton (Hanic et al. 2009; Scholz et al. 2016; Buaya et al. 2017), our data suggest that many marine *Oomycota* are likely pathogenic. If true, *Oomycota* could play an important ecological role in marine environments by constraining primary producer biomass, while contributing to the carbon flow in marine food webs through mechanisms analagous to the mycoloop (Kagami et al. 2014). Moreover, the detection of several *Oomycota* OTUs in only the Bering Sea suggests that lower-latitude *Oomycota* could migrate into the warming Arctic Ocean, thereby interacting with non-coevolved hosts, leading to unforeseeable changes in the communities of primary producers.

The functional gene microarray from under-ice marine sediment in Barrow, Alaska identified a number of genes involved in biogeochemical cycling and parasitism. Some of these biogeochemical cyling genes are known to be involved in carbohydrate metabolism (mannase, oxylose isomerase, and pectate lyase), as well as the degradation recalcitrant materials, such as chitin (chitinase). While the presence of these genes is not surprising, their detection in sediment provides empirical evidence from the Arctic Ocean to support *Oomycota*-mediated carbon cycling. Substantial coupling between benthic and sea ice environments, especially in coastal environments, (Søreide et al. 2013, Gradinger et al. 2009), suggests these processes are also catalyzed in the sympagic system. In addition to common carbon cycling gene products, we detected a number of gene products associated with pathogenicity. Specifically, we detected INF1, which encodes for a secreted protein that can induce a hypersensitive response in plants, thereby causing necrosis, but also confining the pathogen. INF1 was first characterized in *Phytophthora infestans*, the causal agent of potato late blight Surprisingly, we detected high levels of INF1 in seafloor sediment, providing early evidence that INF1 variants are important and evolutionarily conserved proteins in oomycetes. However, the function of INF1 variants in holocarpic organisms is unknown. It is conceivable that pathogenicity of marine oomycetes is similarly complex to terrestrial oomycetes (Gachon et al. 2009). In our preliminary microscopic screening, *Oomycota*-parasitizing diatoms were observed in the Arctic Ocean, but these microscopic observations need to be confirmed with a dedicated systematic screening approach. Future research should focus on exploring the seasonal dynamics of host and associated oomycete parasites, the molecules that interface these biological interactions, and ultimately the proportion of resistant and susceptible variants within these species.

Collectively, the data presented in this study provides a baseline of *Oomycota* diversity, distribution, and putative functioning in the Arctic marine environment that opens the door for future studies to explore the disease ecology of *Oomycota* and to eventually place them into a larger trophic and evolutionary framework.

## CONCLUSIONS

Oomycetes exist throughout the Arctic marine realm and can seasonally comprise > 5% of 18S rRNA amplicon sequence reads. Arctic marine oomycetes parasitize diatoms and encode genes responsible for interfacing virulence and biogeochemical cycling processes. As the Arctic continues to warm, lower-latitude *Oomycota* might migrate into the Arctic Ocean and parasitize non-coevolved hosts.

## Additional file

Additional file 1: Alignment used in this study. (FAS 90 kb)

## Abbreviations

ARA: Average relative abundance
DNA: Deoxyribonucleic acid
HEM: Heterotrophic eukaryotic microbes
OTU: Operational taxonomic unit
PCR: Polymerase chain reaction
rRNA: Ribosomal ribonucleic acid
SD: Standard deviation
V: Variable
